# Effects of high-frequency stimulation and doublets on dynamic contractions in rat soleus muscle exposed to normal and high extracellular [K^+^]

**DOI:** 10.1002/phy2.26

**Published:** 2013-07-15

**Authors:** Katja K Pedersen, Ole B Nielsen, Kristian Overgaard

**Affiliations:** 1 Department of Public Health, Section of Sport Science, Aarhus UniversityAarhus, Denmark; 2 Department of Biomedicine, Aarhus UniversityAarhus, Denmark

**Keywords:** excitability, power, rate of force development

## Abstract

The development of maximal velocity and power in muscle depends on the ability to transmit action potentials (AP) at very high frequencies up to about 400 Hz. However, for every AP there is a small loss of K^+^ to the interstitium, which during intense exercise, may build up to a point where excitability is reduced, thus limiting the intensity of further exercise. It is still unknown how the muscle responds to high-frequency stimulation when exposed to high [K^+^]. Contractile parameters of the muscles (force [*F*], velocity [*V*], power [*P*], rate of force development [RFD], and work) were examined during dynamic contractions, performed in vitro using rat soleus muscles incubated in buffers containing 4 or 8 mmol/L K^+^ and stimulated with constant trains of tetanic or supratetanic frequency or with trains initiated by a high-frequency doublet, followed by tetanic or subtetanic trains. At 4 mmol/L K^+^, an increase in frequency increased *P*_max_ when using constant train stimulation. When stimulating with trains containing high-frequency doublets an increase in 120-msec work was seen, however, no increase in *P*_max_ was observed. At 8 mmol/L K^+^, no differences were seen for either *P*_max_ or 120-msec work when increasing frequency or introducing doublets. In all experiments where the frequency was increased or doublets applied, an increase in *RFD* was seen in both normal and high [K^+^]. The results indicate that stimulation with supratetanic frequencies can improve dynamic muscle contractility, but improvements are attenuated when muscles are exposed to high extracellular [K^+^].

## Introduction

Maintenance of excitability is crucial for muscle function. During intense contractions, however, the maintenance of excitability is adversely affected by a net efflux of K^+^, which occurs in muscle fibers during repeated action potentials. The increased K^+^ efflux can trigger a sequence of events beginning with a rise in [K^+^] in the extracellular fluid, which then leads to depolarization of the membrane potential and induces a slow inactivation of the Na^+^ channels (Ruff 1996), ultimately reducing excitability. Thus, muscle interstitial K^+^ concentrations were shown to approach 10 mmol/L in humans during exercise to exhaustion (Mohr et al. 2004). Studies on isolated rat muscles have shown that such K^+^ concentrations are sufficiently high to decrease muscle fiber excitability and thereby, lead to a loss of force and power (Clausen and Everts 1991; Nielsen et al. 2001; Overgaard et al. 2010).

The efflux of K^+^ from muscle is directly related to the rate of action potential firing. Consequently, increasing the frequency of muscle activation led to increased loss of K^+^ and increased loss of force in isolated rat muscle contracting to fatigue (Clausen et al. 2004). Furthermore, the depolarization might lengthen the refractory period following each action potential, thereby, hampering the ability of the muscles to respond to high-frequency stimulation (Cairns and Dulhunty 1995). Thus, repeated high-frequency activation of muscles could be self-limiting due to rapid loss of fiber K^+^ and fiber depolarization. Still, however, it is not known how the ability of the muscles to respond to high-frequency stimulation is affected by elevated [K^+^]_o_. This problem is particularly important because, in muscles, a high stimulation frequency is necessary for optimal dynamic contractile function, measured as power or velocity. This is exemplified by de Haan (1998), who in rat muscle in situ observed that greater power was developed in dynamic contractions when stimulating with a frequency of 400 Hz compared with other supratetanic frequencies of 120 and 200 Hz, despite the fact that in isometric conditions, the forces developed at these three frequencies were almost identical. The results, therefore, suggested that increasing frequency shifted the force–velocity characteristics of the muscle toward higher force for a given velocity without altering the maximal force producing capacity. In line with this, supratetanic stimulation frequencies increased power in dynamic contractions in an in situ study (Abbate et al. 2000). This study showed that it is the initial stimulation frequency in a contraction, which, for a large part, determines the dynamic contractile characteristics. Thus, only two or three stimulation pulses with a short interpulse interval (doublets or triplets), followed by a train of pulses delivered by a lower frequency was sufficient to increase force of contractions at a given velocity. Interestingly, such doublets and triplets do occur naturally in vivo. For example, in a human study, Van Cutsem et al. (1998) recorded two to three impulses occurring with low interspike intervals corresponding to frequencies of 200–500 Hz in the beginning of a ballistic contraction, and also in humans, Christie and Kamen (2006) recorded motor unit frequencies of 116–714 Hz for two to three pulses at the beginning of dynamic contractions.

Doublets seem to occur in both dynamic (Binder-Macleod 1995; Van Cutsem et al. 1998) and isometric contractions (Bawa and Calancie 1983), at both fast (Van Cutsem et al. 1998) and slow contraction velocities (Bawa and Calancie 1983; Kudina and Churikova 1990). Doublets have been shown to occur in both slow twitch (Van Cutsem et al. 1998; Abbate et al. 2002; Christie and Kamen 2006) and fast twitch motor units (Hennig and Lømo 1985; Van Cutsem et al. 1998; Gorassini et al. 2000). When the doublet is initiating a contraction it is suggested that it contributes to an increase in rate of force development (RFD) and contraction velocity (Van Cutsem et al. 1998; Aagaard et al. 2002; Del Balso and Cafarelli 2007). Based on this, the aim of this study was to investigate dynamic muscle contractile responses to continuous high-frequency stimulations and doublet-initiated stimulations. The experiments were carried out under control conditions with normal (4 mmol/L) extracellular K^+^ concentration and under “near fatigue” conditions where excitability is reduced due to an increased extracellular K^+^ concentration (8 mmol/L). We chose 8 mmol/L extracellular K^+^ in order to simulate a situation where muscle tetanic force was only slightly affected. We hypothesized firstly, that raising the extracellular K^+^ concentration would prevent the muscle from responding with increased power, velocity, and RFD in dynamic contractions when stimulation, delivered as constant frequency trains, was increased from tetanic to supratetanic frequencies. Likewise, we hypothesized that initiating a tetanic or subtetanic pulse train with a doublet of supratetanic frequency would increase power, velocity, and RFD and that this response to doublets would be attenuated by an increased extracellular K^+^ concentration.

The study was performed using electrically stimulated isolated rat soleus muscles incubated at solutions containing different K^+^ concentrations. In these muscles, contractile functions (force [*F*], velocity [*V*], power [*P*], rate of force development [RFD], and initial work) were measured during dynamic contractions elicited by stimulation with different frequency patterns.

## Methods

### Animals, muscle preparation, and buffers

All handling and use of animals complied with Danish animal welfare regulations. All experiments were carried out using soleus muscles from 4-week-old Wistar rats weighing 62–80 g (own breed). The rats were fed ad libitum and were kept at thermostated environmental 21°C with a 12/12 h light/dark cycle. Before experiments, the animals were killed by cervical dislocation followed by decapitation and soleus muscles were dissected out with the proximal end attached to the bone and the distal end with an intact tendon. As standard incubation medium a Krebs–Ringer bicarbonate buffer (4 mmol/L K^+^) was used, containing (mmol/L): 122 NaCl, 25 NaHCO_3_, 2.8 KCl, 1.2 KH_2_PO_4_, 1.2 MgSO_4_, 1.3 CaCl_2,_ and 5.0 d-glucose. In K^+^-enriched buffer (8 mmol/L K^+^) an equivalent amount of Na^+^ was omitted to maintain isoosmolarity. Buffers were equilibrated with a mixture of 95% O_2_ and 5% CO_2_ throughout the experiment. Similar to other studies on both whole muscle and isolated fibers (Segal and Faulkner 1985; Lännergren and Westerblad 1987), the muscles showed an irreversible loss of force if incubated at temperatures above 35°C for intervals long enough to make complete force–velocity curves. For that reason, all experiments were done at 30°C. In all experimental series, the muscles were initially mounted at optimal length for isometric twitch force generation in the standard incubation medium and equilibrated for at least 30 min before starting the experiments.

### Dynamic contractions and experimental protocols

Muscles were mounted on a length/force controlled lever system (model 305; Aurora scientific, Aurora, Ontario, Canada) (for details, see Overgaard et al. 2004). A personal computer generated the signal for electrical stimulation and force/length control of the levers during the contraction through commercial software (DMC v. 4.1.6; Aurora scientific, Aurora, Ontario, Canada), which also allowed for simultaneous sampling of force and length at 1000 Hz. Muscle contractions were evoked via field stimulations using supramaximal constant currents of 0.2 msec duration applied through two platinum plate electrodes. In some experiments, when stimulating with an initiating doublet of high frequency followed by a constant frequency train, a second stimulator (Grass Stimulator, Model S88, Grass Products, Warwick, RI) was connected to the original setup. The DMC v. 4.1.6 software provided the initiating two impulses, and the Grass stimulator provided the subsequent 1.5 sec train of pulses.

**Figure 1:**
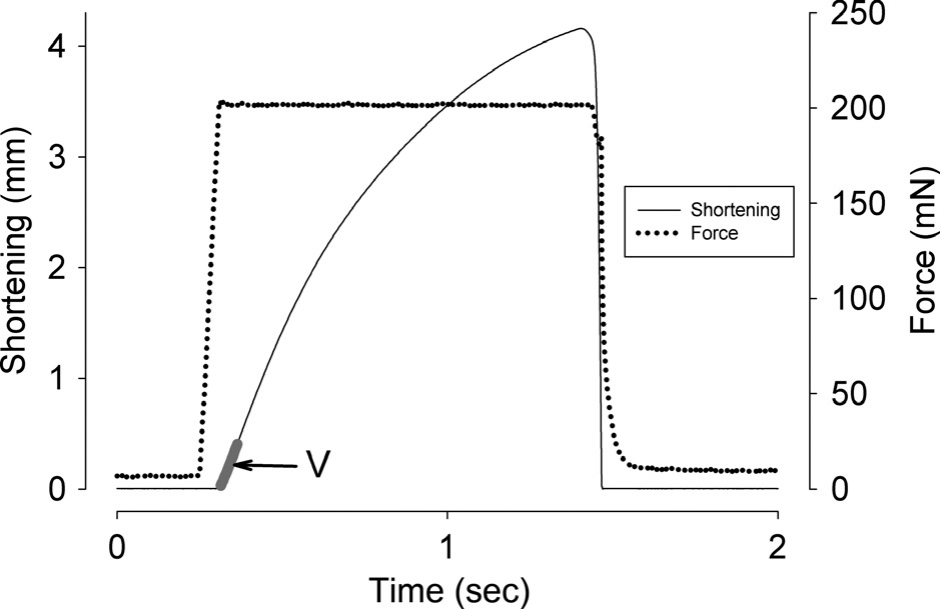
Single contraction protocol for dynamic contractions. The figure shows a sample force trace (dotted line) and shortening trace (solid line) from one contraction protocol (200 mN holding force, constant frequency train) for a soleus muscle. The muscle was stimulated from 0.25 sec, and when the force reached the holding force the muscle started shortening. A 50 msec interval of the shortening curve was used for determination of the shortening velocity (thick line indicated with *V*). Using this method, the highest velocity was determined in each experiment, which was most often at the initiation of the contraction.

To determine force–velocity curves (FV-curves) under different conditions, force and length data were recorded during 7–8 brief contractions that were performed at various holding forces: The muscle was stimulated in a position corresponding to its optimal length, and when the development of force reached the holding force of the lever arm, the muscle began to shorten (Fig. 1). To construct FV-curves, the maximal shortening velocity was in each contraction determined as the maximal shortening over a 50 msec interval, as indicated in Figure 1. After each contraction the relaxed muscle was reextended to its initial length, see Figure 1.

The initial incubation was always standard buffer containing 4 mmol/L K^+^ to establish baseline. After 30 min equilibration, isometric and 6–8 dynamic contractions were performed. Then the muscles were incubated in experimental buffer containing 8 mmol/L K^+^ for at least 60 min and the series of isometric and dynamic contractions were repeated.

To determine relevant stimulation frequencies, a control experiment was conducted where force was measured when muscles were stimulated with frequencies from 1 to 400 Hz after they were positioned isometrically at their optimal length. Stimulation with 60 Hz was sufficient to produce near maximal force (96 ± 2% of the maximal force, *N* = 4). Furthermore, at this frequency the contraction profiles displayed smooth tetani (data not shown). Based on this, 30 Hz (producing 81 ± 8% of the maximal force, *N* = 4) was chosen for a subtetanic stimulation frequency and 60 Hz was chosen for a tetanic stimulation frequency. For supratetanic stimulation, 300 Hz (producing 96 ± 1% of maximal force) was used when stimulating with a constant train, and 245 Hz was used when stimulating with a doublet-containing train.

### Tetanic and supratetanic constant frequency trains

Dynamic contractions were elicited by stimulating the muscles with 1.25 sec constant frequency trains of either 60 or 300 Hz (see Fig. 1). The contractions were repeated eight times using different levels of holding force of the lever arm: 350 mN, 300 mN, 250 mN, 200 mN, 150 mN, 100 mN, 50 mN, and 20 mN. When the muscles were incubated in standard incubation medium, the contractions were separated by 3 min pauses. When at 8 mmol/L K^+^ the pauses were increased to 5 min to take account for the reduced endurance induced by this concentration of K^+^. Isometric force was recorded when stimulating the muscle with 60 Hz with the lever arm at a fixed position corresponding to the optimal muscle length for force production.

To examine whether muscle contractility was preserved throughout the experiment, muscles were transferred back to the standard buffer after ended protocol at 8 mmol/L K^+^ and an isometric contraction of 60 Hz as well as two dynamic contractions of 60 Hz and 300 Hz with the holding force level of 100 mN were performed. The isometric force was 95 ± 3% and power 85 ± 3% (60 Hz) and 98 ± 2% (300 Hz), compared to initial measurements in standard buffer.

### Doublet-containing trains of tetanic and subtetanic frequency

**Figure 2:**
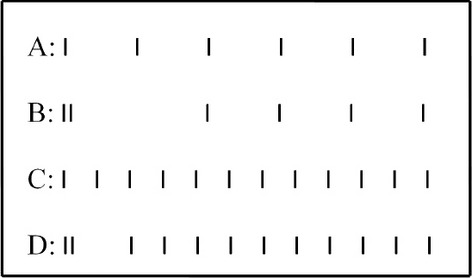
Illustration of the start of a stimulation protocol for normal trains and trains initiated with a doublet. (A) A constant frequency train of 30 Hz. (B) A train initiated with a doublet of 245 Hz followed by a train of 30 Hz. The doublet is effectively the result of transferring the second of the two initiating impulses closer to the first, but maintaining the interval between the first and the third impulse at 66.7 msec. (C) A constant frequency train of 60 Hz. (D) A train initiated with a doublet of 245 Hz followed by a train of 60 Hz, with a 33.3 msec interval between the first and third impulse.

Dynamic contractions were elicited by stimulating the muscles by a train 1.5 sec of 30 Hz, 60 Hz, or by a doublet of 245 Hz, followed by a 1.5 sec train of 30 Hz or 60 Hz. When contractions ended with a train of 30 Hz, the interval between the initial impulse of the doublet and the initial impulse in the subsequent train was 66.7 msec, and when the train was of 60 Hz, the interval was 33.3 msec. Thus, the doublets are the result of transferring the second impulse in a constant frequency train closer to the first impulse in the train, and thereby prolonging the interimpulse interval between the second and the third impulse, see Figure 2. In this way, the number of stimuli was evened out already after the third impulse during a train with doublets compared with a nondoublet train. Also it has been shown that in human motor units, doublets are usually followed by a longer than average interspike interval (Partanen and Lang 1978) The dynamic contractions were repeated six times separated by 3 min pauses, in standard incubation medium, using the holding force levels: 350 mN, 200 mN, 150 mN, 100 mN, 60 mN, and 30 mN. When the muscles were incubated at 8 mmol/L K^+^ the pauses were increased to 5 min as described earlier. Isometric force was recorded as described under constant frequency trains and stimulated with a train of 30, 60 Hz, or by a doublet of 245 Hz with a subsequent train of 30 or 60 Hz, these recordings were also used to create the FV-curves.

An isometric contraction was performed in the beginning and after each protocol in 8 mmol/L K^+^ buffer to check for deterioration of the muscle preparation due to fatigue induced by the stimulation protocol or mechanical slippage (change of length). In the experiment of doublet-containing trains the means for measured force for 30, 60 Hz, 245–30 Hz, and 245–60 Hz, after a stimulation protocol, were respectively 100 ± 0%, 98 ± 0%, 100 ± 0%, and 99 ± 0%, compared to initial force.

### Data analysis

The recorded force and length data were checked and relevant selections extracted using custom made software (SVX, Department of Public Health, Aarhus University, Denmark). For each muscle and for each condition corresponding data points for maximal velocity (*V*) and force (*F*) obtained from the 6–8 dynamic contractions were fitted to the Hill equation:


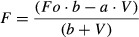


For each muscle and for each condition the parameters a, b, and maximal isometric force (*F*_o_) were obtained. From the equation, maximal velocity (*V*_max_), maximal power (*P*_max_), and curvature (a/*F*_o_) were calculated. Curves were also fitted to a power equation, a variant of the Hill equation:


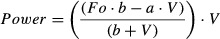


The curve fittings were performed using curve fitting features of the Sigma Plot 11.0 software (Systat software, Inc., Chicago, IL).

The RFD was measured as the maximal rise in force using a rolling average over 10 msec. Work was calculated by the formula:


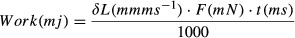


where *ΔL* is muscle shortening, *F* is the holding force, and *t* is the interval over which work is calculated. In all determinations *t* was set to 120 msec to allow time for the stimulation of three impulses when stimulating with a train of 30 Hz, whereby the interval contained both the doublet and the following impulse in the subsequent train of stimuli.

All data are expressed as means ± SEM. The statistical significances of interaction and differences between treatment and frequencies were ascertained using a two-way analyses of variance (ANOVA) for repeated measures followed by a post hoc multiple comparisons analysis of pair wise differences between treatment and between frequencies using a Tukey test as provided by the statistical package in Sigma Plot 11.0. Statistical significance was accepted at *P* < 0.05.

## Results

### Tetanic and supratetanic constant frequency trains

**Figure 3:**
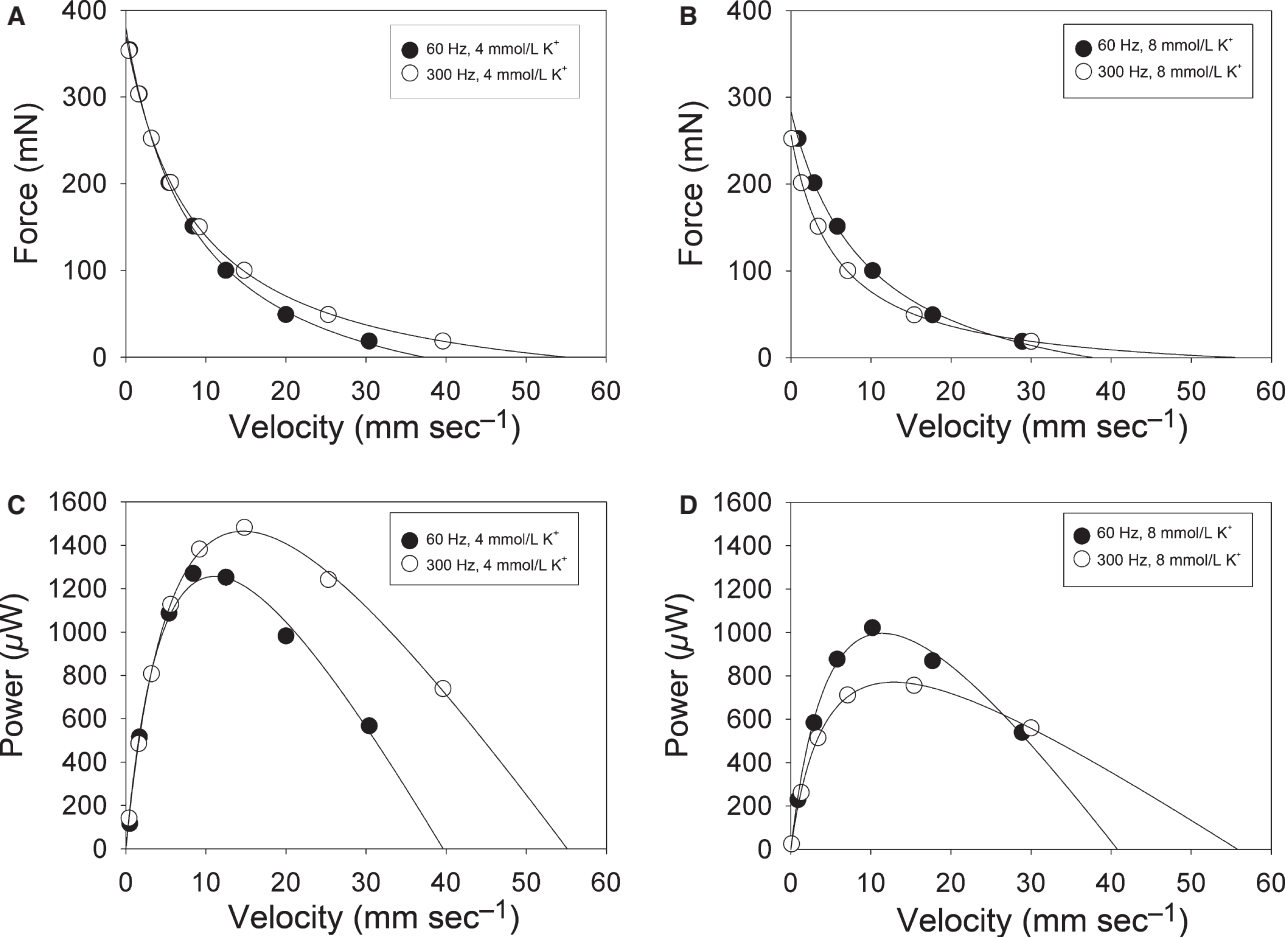
Effect of stimulation frequency and extracellular K^+^ concentration on force–velocity and power–velocity curves from a rat soleus muscle. Force, velocity, and power data for one representative soleus muscle were fitted to Hill equations for force–velocity (A and B) and power–velocity curves (C and D). The muscle was stimulated with a constant frequency train of either 60 or 300 Hz during incubation at 4 mmol/L K^+^ (A and C) or 8 mmol/L K^+^ (B and D).

**Figure 4:**
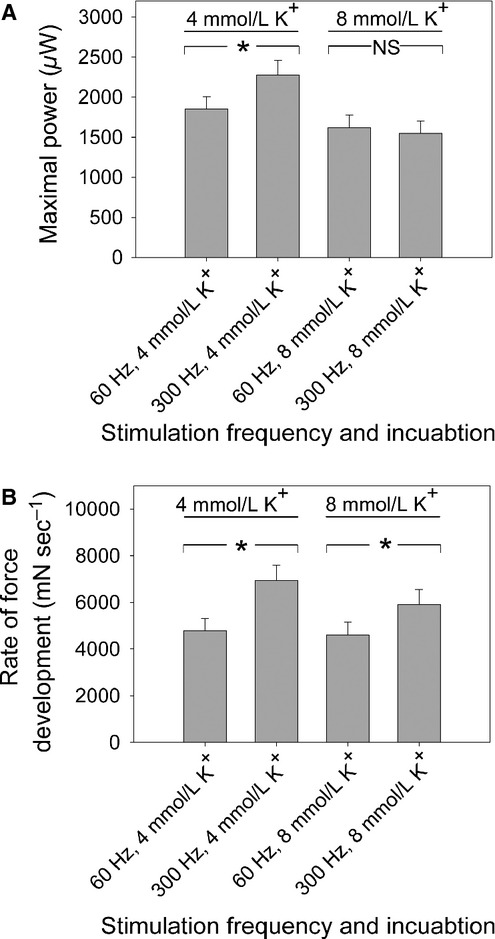
(A) Maximal power (*P*_max_) calculated from parameters of the Hill equation for soleus muscles stimulated with constant frequency trains of 60 and 300 Hz, and incubated at either 4 or 8 mmol/L K^+^. (B) Maximal rate of force development (RFD), measured at a holding force level of 35 g, using a rolling average over 10 msec, for soleus muscles stimulated with constant frequency trains of 60 and 300 Hz, and incubated at either 4 or 8 mmol/L K^+^. All data are expressed as means ± SEM, *n* = 10. *Different from 60 Hz at similar K^+^ concentration (*P* < 0.05). ^NS^Not significantly different from 60 Hz at similar K^+^ concentration.

In this series of experiments, force–velocity curves were obtained for muscles stimulated with 1.25 sec trains of pulses at tetanic (60 Hz) and supratetanic (300 Hz) frequencies during incubation at 4 and 8 mmol/L K^+^ (Fig. 3). In isometric contractions, this led to a force production that reached a plateaued after ∼0.5 sec and remained stable throughout the contractions (data not shown). Force profile for the dynamic contractions is shown in Figure 1. From these experiments, values for force, velocity, and power were determined and fitted to Hill equations for force–velocity and power–velocity curves. As shown in Figure 3A and B this demonstrated a tendency for the maximal shortening velocity to increase when the stimulation frequency was increased from 60 to 300 Hz. This effect was seen at both 4 and 8 mmol/L K^+^. In contrast, a tendency for an increase in *P*_max_ with the increase in stimulation frequency observed at 4 mmol/L K^+^ (Fig. 3C) was at 8 mmol/L K^+^ reversed to a decrease (Fig. 3D). These effects are summarized in Figure 4 that shows that at 4 mmol/L K^+^, the muscles produced a significantly higher *P*_max_ and RFD, when stimulated with 300 Hz trains than when stimulated with 60 Hz trains. Thus, *P*_max_ increased by 22%, and RFD increased by 45% when increasing stimulation frequency from 60 to 300 Hz. However, when muscles were incubated at 8 mmol/L K^+^, increasing frequency from 60 to 300 Hz did not produce higher *P*_max_ (Fig. 4A) but RFD was still significantly increased by 28% at 300 Hz compared with 60 Hz (*P* < 0.05, Fig. 4B). Likewise, stimulation with 300 Hz increased *V*_max_ compared with stimulation with 60 Hz, both at 4 and 8 mmol/L K^+^ (Table 1).

**Table 1:** Effect of 4 and 8 mmol/L K^+^ on dynamic contractions in soleus muscles stimulated with constant frequency trains of 60 or 300 Hz

Treatment	*F*_o_ (mN)	Curvature (*a/F*_o_)	*V*_max_ (mm sec^−1^)	*F* at *P*_max_ (mN)
4 mmol/L K^+^ 60 Hz	452 ± 39	0.19 ± 0.03	56 ± 4	118 ± 8
4 mmol/L K^+^ 300 Hz	422 ± 20^1^	0.19 ± 0.01^2^	66 ± 3^1^	118 ± 6^2^
8 mmol/L K^+^ 60 Hz	363 ± 29	0.22 ± 0.02	51 ± 3	108 ± 7
8 mmol/L K^+^ 300 Hz	324 ± 29^1^	0.18 ± 0.04^2^	67 ± 16^1^	88 ± 6^1^

Isometric force (*F*_o_), curvature (*a/F*_o_), maximal velocity (*V*_max_), and the calculated force at maximal power (*F* at *P*_max_) were obtained from the Hill equation made for each muscle by fitting corresponding data for force and velocity (see Fig. 4 for examples). All data are expressed as means ± SEM; *n* = 10.

1 Different from 60 Hz at a similar K^+^ concentration (*P* < 0.05).

2 Not significantly different from 60 Hz at similar K^+^ concentration.

As power is the product of force and velocity, the observation that incubation at 8 mmol/L K^+^ prevented the frequency dependent increase in *P*_max_ despite an increase in shortening velocity, indicated that the force during dynamic contraction was reduced at high frequency. To examine this, the force at maximal power (*F* at *P*_max_) was calculated. As shown in Table 1, this calculation revealed that whereas the *F* at *P*_max_ was unaffected by an increase in the stimulation frequency from 60 to 300 Hz in muscles at 4 mmol/L K^+^, it was significantly lowered in muscles at 8 mmol/L K^+^. The effect of K^+^ per se on power and force can also be evaluated when comparing values for 4 and 8 mmol/L K^+^ combined with the same stimulation frequency. This showed that when increasing [K^+^] from 4 to 8 mmol/L, *P*_max_ decreased by 13% for 60 Hz and by 32% for 300 Hz, and *F* at *P*_max_ decreased by 8% for 60 Hz and by 25% for 300 Hz, *P* < 0.05.

### Doublet-containing trains of tetanic and subtetanic frequency

We went on to examine whether it was possible to achieve improved dynamic contractility by introducing doublets with supratetanic frequency (245 Hz) into a stimulation train with a lower frequency of either 60 Hz (tetanic) or 30 Hz (subtetanic).

When a 245 Hz doublet is introduced in a 60 Hz train at 4 mmol/L K^+^, the dynamic contractile parameters (*P*_max_, *V*_max_, *F*_o_) were mostly unaffected compared to a constant train of 60 Hz. In fact, we observed surprisingly that the doublet-containing train had slightly, but significantly, lower *V*_max_ and *P*_max_ than the constant train of 60 Hz (Table 2). However, a significant increase by 30% in RFD for the 60 Hz doublet-containing train was observed compared with the constant train of 60 Hz (Table 2). An initiation with a doublet in a 30 Hz train did not give any increase in *F*_o_, compared with a constant train of 30 Hz. However, there was an increase in *V*_max_ by 8% and RFD by 68%, when muscles were stimulated with a doublet of 245 Hz followed by a subtetanic train of 30 Hz, compared with a constant train of 30 Hz. However, even though this increase was significant, there was no significant increase in *P*_max_ (Table 2).

**Table 2:** Effect of elevated extracellular K^+^ and the introduction of an initial doublet of 245 Hz into constant frequency trains of 60 or 30 Hz on dynamic contractions in soleus muscles

Treatment	*F*_o_ (mN)	*V*_max_ (mm sec^−1^)	*P*_max_ (μWatt)	Curvature (*a/F*_o_)	RFD (mN sec^−1^)
4 mmol/L K^+^ 60 Hz	471 ± 10	43 ± 1	2052 ± 69	0.29 ± 0.01	4871 ± 157
4 mmol/L K^+^ 245-60 Hz	452 ± 10^1^	40 ± 0.7^2^	1935 ± 49^2^	0.31 ± 0.02^1^	6344 ± 216^2^
4 mmol/L K^+^ 30 Hz	412 ± 10	26 ± 0.6	1316 ± 39	0.43 ± 0.03	4164 ± 167
4 mmol/L K^+^ 245-30 Hz	422 ± 10^1^	28 ± 0.9^3^	1326 ± 39^1^	0.34 ± 0.02^3^	6982 ± 226^3^
8 mmol/L K^+^ 60 Hz	442 ± 10	44 ± 1	1984 ± 69	0.29 ± 0.01	5273 ± 147
8 mmol/L K^+^ 245-60 Hz	432 ± 10^1^	42 ± 1^1^	1876 ± 59^2^	0.29 ± 0.01^1^	6737 ± 324^2^
8 mmol/L K^+^ 30 Hz	393 ± 10	28 ± 0.9	1277 ± 39	0.38 ± 0.02	4733 ± 147
8 mmol/L K^+^ 245-30 Hz	403 ± 10^1^	29 ± 1^1^	1267 ± 49^1^	0.33 ± 0.02^1^	6786 ± 344^3^

Isometric force (*F*_o_), maximal velocity (*V*_max_), maximal power (*P*_max_), and curvature calculated from the Hill equation and rate of force development (RFD) from soleus muscles incubated at 4 or 8 mmol/L K^+^ and stimulated with constant frequency trains of 60 or 30 Hz with or without an initial doublet of 245 Hz. All data are expressed as means ± SEM. All *P* < 0.05; *n* = 10.

1 Not significantly different from a constant train of same frequency at similar K^+^ concentration.

2 Different from a 60 Hz train at similar K^+^ concentration.

3 Different from a 30 Hz train at similar K^+^ concentration.

At 8 mmol/L K^+^, introduction of doublets into 30 or 60 Hz trains showed the same pattern as at 4 mmol/L K^+^, where it had a minimal effect. Thus, similar to incubation at 4 mmol/L K^+^, at 8 mmol/L K^+^ there was a slight decrease in *P*_max_ for a doublet-containing train of 60 Hz compared with a constant train of 60 Hz (Table 2). However, RFD was still significantly increased by 28% and by 43% for 60 Hz and 30 Hz trains initiated with a doublet (Table 2).

**Figure 5:**
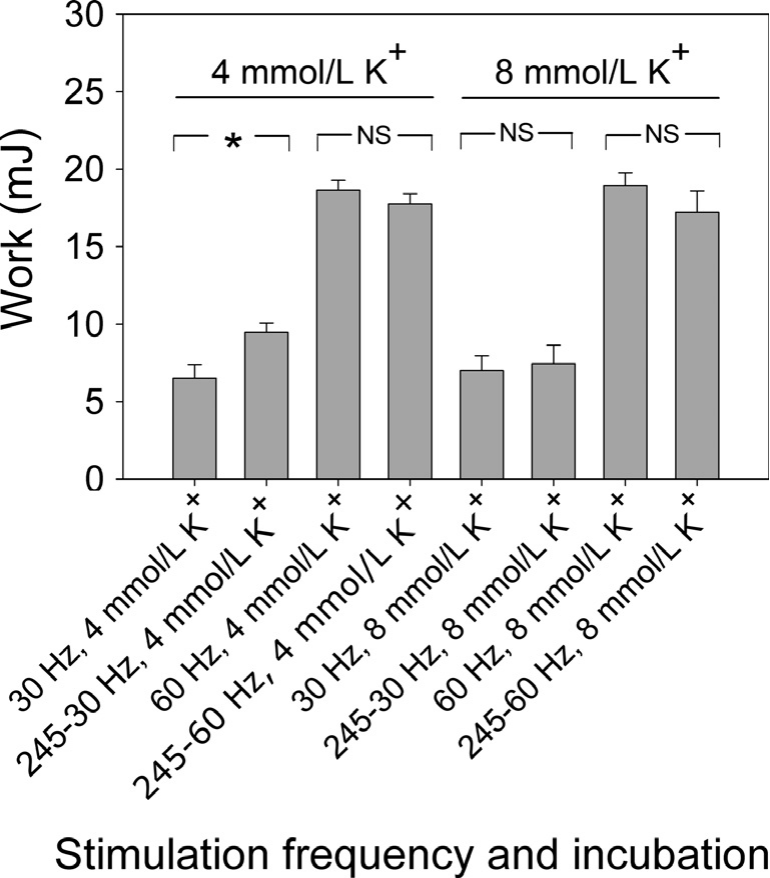
Work produced by soleus muscles during the first 120 msec of a contraction when stimulated with constant frequency trains of 60 or 30 Hz with or without an initial doublet of 245 Hz and incubated at 4 or 8 mmol/L K^+^ (120-msec work). All data are expressed as mean ± SEM. *Different from a train of 30 Hz at similar K^+^ concentration. ^NS^Not significant different from a constant train of same frequency at similar K^+^ concentration. All *P* < 0.05; *n* = 10.

To evaluate the importance of extracellular K^+^ and the inclusion of a doublet in the stimulation trains for the ability of the muscle to perform work over a brief period of time (e.g., corresponding to the ground contact time during running), we measured the work produced in the first 120 msec from the beginning of the stimulation (120-msec work). When introducing a doublet into a train of tetanic frequency (60 Hz), no change was seen in 120-msec work at intermediary forces (150 mN) (Fig. 5). However, when introducing a doublet into a train of subtetanic frequency (30 Hz) a significant improvement of 45% compared to a train of 30 Hz, was seen. At 8 mmol/L K^+^ no improvement was seen for 120-msec work when introducing a 245 Hz doublet into either a 30 or 60 Hz train.

When comparing 4 mmol/L K^+^ and 8 mmol/L K^+^ a significant decrease by 27% was seen in 120-msec work when changing incubation from 4 to 8 mmol/L K^+^ for a doublet-containing train of 30 Hz, P < 0.05.

## Discussion

This study demonstrated that increasing the stimulation frequency from 60 Hz to the supratetanic range led to a significant increase in the dynamic contractile parameters (RFD, *V*_max_, and *P*_max_) in muscles incubated at 4 mmol/L K^+^. This improvement in dynamic contractility occurred despite the fact that the maximal tetanic force at 60 Hz was similar to the maximal isometric force at 300 Hz. Similarly, when a doublet-containing stimulus train was applied to muscles, the initial work (120-msec work) was increased compared to muscles receiving a constant frequency train of subtetanic frequency. However, the positive effects of the high-frequency stimulation on power and work were markedly reduced in muscles incubated at 8 mmol/L K^+^.

### Effects of high-frequency stimulation on muscle power and velocity at 4 mmol/L K^+^

In the present study, rat soleus muscles achieved near maximal force when stimulated with a frequency of 60 Hz. Despite this, an increase in stimulation frequency to above 60 Hz significantly increased the dynamic contractile parameters *V*_max_, *P*_max_, and RFD (Table 1 and Fig. 4A and B). Likewise, Abbate et al. (2000) found that a constant frequency train of 200 Hz produced a larger increase in power, compared with trains of 80 and 120 Hz. The experiments were performed in situ on rat gastrocnemius muscles using velocity as the independent parameter. A similar result, where velocity also was used as the independent parameter, was seen by de Haan (1998), who observed that at high shortening velocity (250 mm sec^−1^) a stimulation frequency of 400 Hz or more was required to produce maximal power output. Although the determinations of *P*_max_ by Abbate et al. (2000) and de Haan (1998) were based on constant velocity experiments whereas our determinations of *P*_max_ were based on constant force contractions, their results are consistent with our observation that an increase in the stimulation frequency for a constant train from 60 to 300 Hz increased *P*_max_.

### High-frequency stimulation at 8 mmol/L K^+^

In order for the muscle membrane to respond adequately to high-frequency stimulation, the absolute refractory time of the muscle fibers for generating action potential has to be shorter than the interimpulse interval. In this respect, it has been shown that the time needed for repolarization at end of the action potential and refractory time of muscle fibers increases when they become depolarized be elevated extracellular K^+^ (Nielsen et al. 2004; Kuwabara et al. 2007; Macdonald et al. 2008), which reduces the ability of the muscles to fire action potentials at high frequency (Macdonald et al. 2008). It can be envisaged, therefore, that as the muscle fibers depolarize during high intensity exercise because of an increase in extracellular [K^+^] the responsiveness of muscles to excitation becomes compromised especially at high stimulation frequencies. This notion is supported by two observations: Firstly, the exposure of rat soleus muscles to 8 mmol/L extracellular K^+^ has little effect on maximal tetanic force reached during the first second of 60 Hz stimulation but greatly increases fatigue development if the stimulation is continued for several seconds (Clausen and Nielsen 2007). Secondly, Bigland-Ritchie et al. (1979) found that in humans the decrease in force development during prolonged, exhausting stimulation protocol of a constant 80 Hz frequency train was partly recovered immediately after decreasing the stimulation frequency to only 20 Hz. Together these observations strongly indicate that the ability of muscle fibers to respond to high-frequency stimulation becomes reduced if the muscle fibers are depolarized by elevated extracellular K^+^. In agreement with this, we here show that an increase in extracellular K^+^ to 8 mmol/L completely abolished the improvement on *P*_max_ observed in muscles at 4 mmol/L when the stimulation frequency was increased from 60 Hz tetanic to 300 Hz supratetanic (Fig. 4A). In addition to this, incubation of the muscles at 8 mmol/L K^+^ caused a small but significant reduction in *F*_o_ and a more pronounced reduction in *F* at *P*_max_ and 120-msec work. In our experiments muscles were stimulated briefly (1.25 sec) with 3 to 5 min intervals. It is likely that a small further increase of K^+^ may occur in the interstitium during these brief contractions, but this seemed to have no measurable consequences for muscle force production as isometric contraction force reached a plateau after ∼0.5 sec in a tetanus and remained stable throughout the remainder of the 1.25 sec of contractions (data not shown). As for the dynamic contractile properties, these were based on measurements early in the contractions where only minor excursions in interstitial K^+^ compared to the buffer level would have occurred. One important consequence of the described effect of 8 mmol/L extracellular K^+^ is that accumulation of K^+^ in the interstitium may be more important for muscle fatigue than previously indicated by evaluations based on measurements of maximal isometric force. Thus, studies on isolated rat muscles show that extracellular K^+^ has to be elevated to 10 to 15 mmol/L to significantly depress maximal isometric force (Nielsen et al. 2001; Hansen et al. 2005; de Paoli et al. 2007; for review of earlier literature, see Nielsen and Overgaard 1996). As this level of interstitial K^+^ only has been observed in humans after intense leg exercise to exhaustion (Nordsborg et al. 2003; Mohr et al. 2004), the high concentrations of extracellular K^+^ necessary to depress force in rats muscles could indicate that elevated extracellular K^+^ only is relevant for fatigue development at very high exercise intensities. In contrast to this, the observation in the present study that dynamic muscle function may be compromised even at 8 mmol/L extracellular K^+^ points to the notion that elevated extracellular K^+^ may play a role in fatigue even at moderate exercise intensities, where the built-up of interstitial K^+^ is less pronounced.

### Effects of doublet-containing trains at 4 and 8 mmol/L K^+^

A number of studies indicate that doublets can improve isometric contractile function in human and animal muscles (Abbate et al. 2002; Bentley and Lehman 2005). Previously, the mechanism behind the effects of doublets were proposed to be due to increased intracellular Ca^++^ (Garland and Griffin 1999), but in 2002, Abbate et al., demonstrated that the increase in Ca^++^ following triplet stimulation was too short lasting to account for the increased force in single muscle fibers (Abbate et al. 2002). Thus, the mechanism behind doublet/triplet induced improvement in contractility seems to involve an initial brief increase in intracellular Ca^++^ followed by a period of increased Ca^++^ sensitivity of the contractile apparatus. This mechanism is exploited in motor programs of humans and animals, where high-frequency doublet or triplets are observed at the beginning of voluntary dynamic contractions (Van Cutsem et al. 1998; Christie and Kamen 2006). These doublets occur with interimpulse intervals corresponding to frequencies of 116–714 Hz. In light of this, we found it interesting to ask, whether it was possible to achieve dynamic contractile improvements using doublets in the beginning of a stimulation train? Somewhat, contrary to our expectations, we observed a slightly decreased *V*_max_ and *P*_max_, when introducing a high-frequency doublet into a stimulation train of 60 and 30 Hz, however, the RFD was improved. In agreement with those results, Binder-Macleod (1995) found that a train initiated by a doublet decreased the force development, compared to a constant frequency train in unfatigued human potentiated quadriceps femoris muscle which was stimulated. In contrast stimulation with the doublet-initiated train in an exhausted muscle increased force development, compared to the constant frequency train (Binder-Macleod 1995). Our results did not show a similar improvement in contractile force or power, neither at 4 nor at 8 mmol/L K^+^. Nevertheless, we did see an increase in RFD in both normal and high potassium, when introducing a doublet in a constant frequency train, compared to a constant frequency train, which was in agreement with results observed by Bentley and Lehman (2005). They showed on the human m. flexor carpi radialis, that a train of 20 Hz initiated with a doublet, increased the rate of force rise compared to a constant 20 Hz train, both under normal conditions and in muscles experiencing low-frequency fatigue.

RFD increased with an increase in frequency and this effect was not compromised by the high K^+^ in our experiment, which means that a doublet has an effect on how fast the muscle is capable of developing force, but not on how much force is developed, as no increases with increasing frequency were seen for maximal *F* in the experiments. The fast development of force is very important when performing time-limited tasks, whether it is the restoration of balance when experiencing a sudden postural perturbation, or performance of an athletic task.

Under many working conditions, there is only a certain amount of time for the muscle to perform a given amount of work. For example, a study by Chapman et al. (2012) on elite male and female runners shows that a decrease in ground contact time for the foot is seen when running speed increases. The running velocities, matched competitive running speeds achieved by high-level distance runners in events ranging from 1500 m to a marathon. The measured ground contact time was approximately 120 to 180 msec. As the contact time decreases, the demands for the work performed when the foot is on the ground increases. In this study, we investigated if the implementation of a doublet in a constant frequency train could have an increasing effect on 120-msec work. This was not the case when a doublet of 245 Hz were introduced in a 60 Hz train, compared to a constant train of 60 Hz, neither at 4 nor 8 mmol/L K^+^. But when we induced a doublet of 245 Hz in a constant train of 30 Hz we did observe an increase in 120-msec work, compared to a constant train of 30 Hz, when incubated at 4 mmol/L K^+^. It can be argued that this type of stimulation pattern, where a doublet is followed by subtetanic train, is similar to what is observed in vivo. Thus, Gorassini et al. (2000) observed in rat muscles that when a doublet occurred it was a common occurrence, that the prolonged interimpulse between the doublet and the following train produced a frequency that “undershot” all the other frequencies in the train. This supports the notion that in vivo it is more likely to see a doublet followed by a subtetanic train, than followed by a tetanic train.

If our results were to be evaluated in a sporting context, the interpretation could be that during intervals with low work intensity where extracellular K^+^ remains low, the muscles would be able to respond with a higher power or work output when stimulated at supratetanic frequency, corresponding to the increase in *P*_max_ (for constant frequency trains) and 120-msec work (for doublet-containing trains of 30 Hz) induced in the present study by supratetanic stimulation at 4 mmol/L K^+^. During intervals with higher work intensity, however, where extracellular K^+^ rises, the same type of stimulation may produce a smaller contractile response when jumping and running, indicating fatigue (Fitts 1994). In the context of the present study this corresponds to the observation that *P*_max_ and 120-msec work were not increased with supratetanic frequencies at 8 mmol/L K^+^. Despite this, activation of muscles at elevated extracellular K^+^ with supramaximal frequencies would not be entirely without effect, as it would still produce an increase in RFD. This notion is based on the observation that for all experiments at 4 and 8 mmol/L K^+^, muscles responded with a faster increase in force when stimulated at supramaximal frequency.

## Conclusion and perspectives

The present study shows that stimulation with supratetanic frequencies can improve dynamic contractility of a muscle. But this improvement is compromised when muscles are exposed to high extracellular K^+^ concentration. Even though an increased frequency produced an increase in work and power during normal conditions, there were no effects on work and power of increasing frequency when muscles were exposed to a high potassium concentration. The only parameter which increased with increasing frequency and maintained this increase in high potassium was RFD. In perspective, as supratetanic frequency is rarely or never maintained for much more than two to three pulses in naturally occurring activation patterns, it could be speculated that the doublet-initiated activation patterns evolved to retain some of the contractile advantages of high-frequency stimulation while minimizing the loss of K^+^ and ensuing fatiguing consequences at the same time. This possibility remains to be examined.
